# Supplementary material for: Light Emission from Fe2+-EGTA-H2O2 System Depends on the pH of the Reaction Milieu within the Range That May Occur in Cells of the Human Body
Source: Molecules. 2024 Aug 25;29(17):4014. doi: 10.3390/molecules29174014 (PMC11396423; doi:10.3390/molecules29174014)
Supplement: Supplementary file 1 [file molecules-29-04014-s001.zip › molecules-3116086-supplementary.pdf]

Article

Light Emission from Fe^{2+}-EGTA-H_2O_2 System Depends on the pH of the Reaction Milieu within the Range That May Occur in Cells of the Human Body

Krzysztof Sasak [1], Michal Nowak [2], Anna Wlodarczyk [3], Agata Sarniak [4], Wieslaw Tryniszewski [5] and Dariusz Nowak [4,*]

[1] Department of Medical Imaging Techniques, Medical University of Lodz, Lindleya 6, 90-131 Lodz, Poland; krzysztof.sasak@umed.lodz.pl
[2] Radiation Protection, University Hospital No. 2, Medical University of Lodz, Zeromskiego 113, 90-549 Lodz, Poland; m.nowak@skwam.lodz.pl
[3] Department of Sleep Medicine and Metabolic Disorders, Medical University of Lodz, Mazowiecka 6/8, 92-215 Lodz, Poland; anna.wlodarczyk@umed.lodz.pl
[4] Department of Clinical Physiology, Medical University of Lodz, Mazowiecka 6/8, 92-215 Lodz, Poland; agata.sarniak@umed.lodz.pl
[5] Department of Radiological and Isotopic Diagnostics and Therapy, Medical University of Lodz, Zeromskiego 113, 90-549 Lodz, Poland; wieslaw.tryniszewski@umed.lodz.pl
* Correspondence: dariusz.nowak@umed.lodz.pl; Tel.: +48-42-2725656; Fax: +48-42-2725652

Supplementary Material

Table S1
Ultra weak photon emission (UPE) from Fe^{2+}- EGTA – H_2O_2 system and appropriate controls depending on the pH of reaction milieu

Sample	pH of reaction milieu							
	6.0	6.2	6.4	6.6	6.8	7.0	7.2	7.4
Fe^{2+}-EGTA-H_2O_2	2761±169 (2767;180)	2696±296 (2674;345)	4300±330 (4357; 348)	4698±583 (4557;1062)	4651±410 (4756;651)	3951±370 (3961;504)	3780±373 (3704;295)	3946±558 (3745;465)
Fe^{2+}-EGTA-H_2O_2 *	2197±170 (2168;118)	2061±340 (1991;358)	3705±281 (3688;198)	4207±586 (4006;1069)	4004±387 (4144;605)	3326±352 (3357;527)	3074±371 (2998;332)	3023±652 (2983;544)
Fe^{2+}-H_2O_2	636±94 (661;184)	758±101 (799;164)	803±100 (802;150)	620±70 (600;71)	747±127 (710;151)	717±92 (766;157)	826±76 (831;127)	822±120 (854;182)
EGTA-H_2O_2	597±90 (598,171)	616±86 (648;150)	593±85 (625:165)	493±44 (492;68)	646±129 (698;221)	623±125 (616;119)	680±70 (686;131)	727±173 (702;161)
H_2O_2 alone	535±40 (533;75)	655±86 (672;119)	600±87 (615;131)	500±48 (498;31)	656±111 (712;212)	673±23 (670;32)	668±60 (711;101)	782±190 (754;49)
Fe^{2+} - EGTA	554±64 (529;96)	651±68 (669;85)	579±88 (619;162)	512±83 (493;68)	638±93 (688;163)	614±75 (617;131)	737±157 (714;84)	754±137 (763;85)
Medium alone	563±61 (540;84)	694±159 (680;84)	595±73 (642;122)	491±47 (486;72)	647±34 (647;46)	625±64 (651;105)	706±77 (695;98)	923±624 (758;103)

Results expressed as mean and standard deviation and (median; interquartile range) were obtained from 9 separate repetitions. UPE was expressed in relative light units (RLU) *- These results represent the ΔUPE = (UPE of Fe^{2+}-EGTA-H_2O_2 - UPE of medium alone). Fe^{2+} - H_2O_2 – incomplete system I; EGTA-H_2O_2 - incomplete system II

Table S2

Effect of pH of reaction milieu on ratios of UPE of Fe^{2+}-EGTA-H_2O_2 to noise, increment in UPE of Fe^{2+}-EGTA-H_2O_2 (ΔUPE) to noise and UPE of Fe^{2+} -H_2O_2 to noise

Sample	Ratio	pH of reaction milieu							
		6.0	6.2	6.4	6.6	6.8	7.0	7.2	7.4
Fe^{2+} - EGTA – H_2O_2	UPE to noise	5.0±0.6 (4.7;0.7)	4.0±0.8 (4.0;0.4)	7.3±0.6 (7.1;1.1)	9.7±1.5 (9.3;2.3)	7.2±0.5 (7.3;0.5)	6.4±0.7 (6.4;0.6)	5.4±0.7 (5.6;0.9)	5.0±1.5 (4.9;0.9)
	Δ UPE to noise	4.0±0.6 (3.7;0.7)	3.0±0.8 (3.0;0.4)	6.3±0.6 (6.1;1.1)	8.7±1.5 (8.3;2.3)	6.2±0.5 (6.3;0.5)	5.4±0.7 (5.4;0.6)	4.4±0.7 (4.6;0.9)	4.0±1.5 (3.9;0.9)
Fe^{2+} - H_2O_2	UPE to noise	1.1±0.2 (1.1;0.2)	1.1±0.2 (1.1;01)	1.3±0.1 (1.4;0.1)	1.3±0.1 (1.2;0.1)	1.1±0.1 (1.1;0.2	1.2±0.1 (1.2;0.1)	1.2±0.1 (1.2;0.1)	1.1±0.3 (1.1;0.1)

Results expressed as mean and standard deviation and (median; interquartile range) were obtained from 9 separate repetitions. Noise is defined as UPE of medium alone.

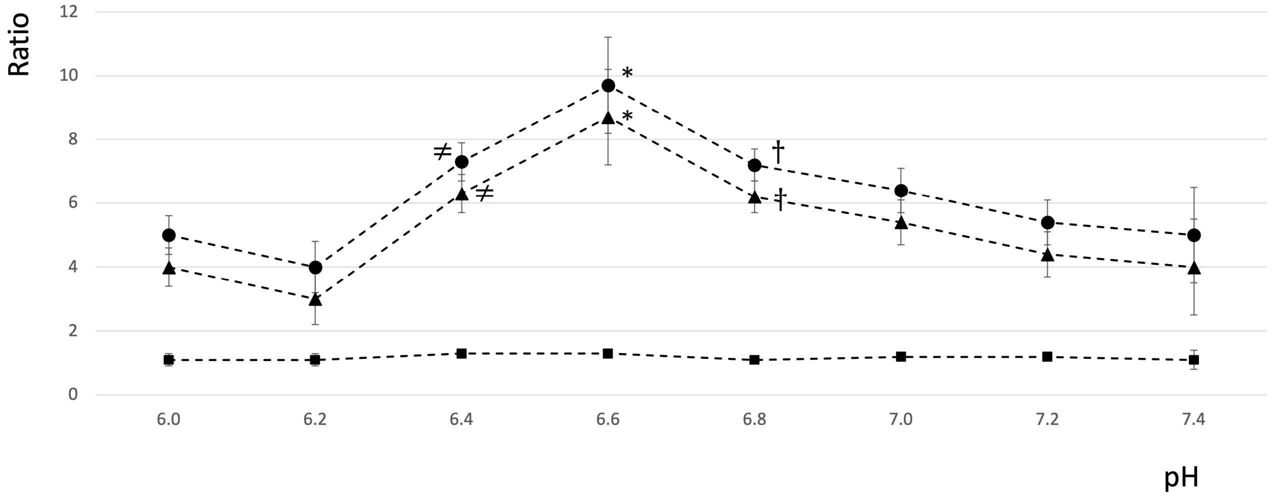

Figure S1. Effect of pH of reaction milieu on UPE (ultra weak photon emission) (-●-) and ΔUPE (UPE minus baseline) (-▲-) of Fe^{2+}-EGTA- H_2O_2 system, Fe^{2+}-H_2O_2 (-♦-) and noise (buffer alone- baseline) (-■-). * - significantly different from corresponding values noted for pH = 6.0, 6.2, 7.0, 7.2 and 7.4 – $p < 0.05$. ┼ - significantly different from corresponding values noted for pH = 6.0, 6.2, 6.4, 7.0, 7.2 and 7.4 – $p < 0.05$. ≠ - significantly different from corresponding values noted for pH = 6.0, 6.2 and 7.2 – $p<0.05$. Each point represents the mean ± SD of nine series of separate experiments.
